# Multiple Major Aortopulmonary Collateral Arteries With Tetralogy of Fallot and Other Congenital Cardiac Disorders Detected in the Fourth Decade: A Report of a Rare Case

**DOI:** 10.7759/cureus.63194

**Published:** 2024-06-26

**Authors:** Isha Sahai, Benumadhab Ghosh, Vaibhav Raut, Vaibhav Mahalle, Gajendra Agrawal

**Affiliations:** 1 Cardiology, Jawaharlal Nehru Medical College, Datta Meghe Institute of Higher Education and Research, Wardha, IND

**Keywords:** malaligned vsd, absent left subclavian artery, right aortic arch, digeorge syndrome, tetralogy of fallot, hypoplastic pulmonary arteries, coronary mapca, mapca

## Abstract

The occurrence of MAPCAs (major aortopulmonary collateral arteries) with TOF (tetralogy of Fallot) and bilateral hypoplastic pulmonary arteries together is a rare condition. Patients are typically middle-aged men who usually present with acute signs of cardiac manifestations. The anomalies have survival up to the fourth decade of life and are fraught with clinical challenges. Additionally, various congenital syndromic associations, such as DiGeorge syndrome, are associated with these anomalies. We report an extremely rare case of a 41-year-old male who came with complaints of chest pain, dyspnea on exertion, and headaches. The patient had a previous history of tuberculosis and a rare combination of MAPCAs with TOF and bilateral hypoplastic pulmonary arteries, with a right-sided aortic arch with an aplastic left subclavian artery. The importance of the case comes from the need to perform surgery on a middle-aged male who was completely asymptomatic prior to this.

## Introduction

Tetralogy of Fallot (TOF) is a common cyanotic type of congenital heart disease (CHD) that consists of ventricular septal defect (VSD), right ventricular hypertrophy, and right ventricular outflow tract obstruction, overriding of the aorta [1]. Pulmonary atresia (PA) is a rare variant of TOF, and major aortopulmonary collateral arteries (MAPCAs) are the main dependable circulatory adjuvants for pulmonary circulation in patients with PA and TOF. The combination of MAPCAs with TOF and PA is the most extreme type of TOF [1]. The 22q11.2 deletion (del22q11), also known as DiGeorge syndrome, occurs in approximately 13% of cases with TOF. This deletion is found in over 30% of individuals with TOF, accompanied by PA and MAPCAs [2-4]. An increased risk of morbidity and mortality is thought to exist in patients with TOF with the association of genetic syndromes [5]. In the more intricate subset of TOF linked with MAPCAs, the presence of del22q11 is linked to elevated mortality rates, as reported, regardless of the extent of pulmonary artery hypoplasia. The existence of a right-sided aortic arch with an aberrant left subclavian artery is very infrequent. Studies have shown a prevalence of 0.01%-0.1% of the general population, as in our patient. Although a right aortic arch with a mirror image of the separation of the head and neck vessels typically does not induce any physiological cardiovascular impacts, it may coincide with other congenital heart anomalies [6]. Anomalies in the branching pattern and orientation of the aortic arch are linked to various congenital heart anomalies, such as TOF and truncus arteriosus (TA), and chromosomal aberrations, such as in DiGeorge syndrome [7].

In this report, we present a case of a middle-aged male who presented with chest pain and breathlessness and was found to have multiple congenital cardiac conditions. The report details the subsequent evaluation and surgical correction performed.

## Case presentation

A 41-year-old male presented to a tertiary care hospital in the cardiac outpatient department (OPD) with complaints of chest pain, breathlessness (New York Heart Association (NYHA) grade 3), and an on-and-off headache for a month. The patient was diagnosed with polycythemia (Hb 22 g/dL) one and a half months ago. He has a documented case of pulmonary tuberculosis, diagnosed five years ago, and has successfully completed a six-month course of anti-tubercular drug therapy. There is no concurrent presence of hypertension or diabetes mellitus. The patient was advised to undergo diagnostic procedures including a chest X-ray, electrocardiography (ECG), computed tomography, aortogram, and angiography. There is no significant family history.

**Figure 1:**
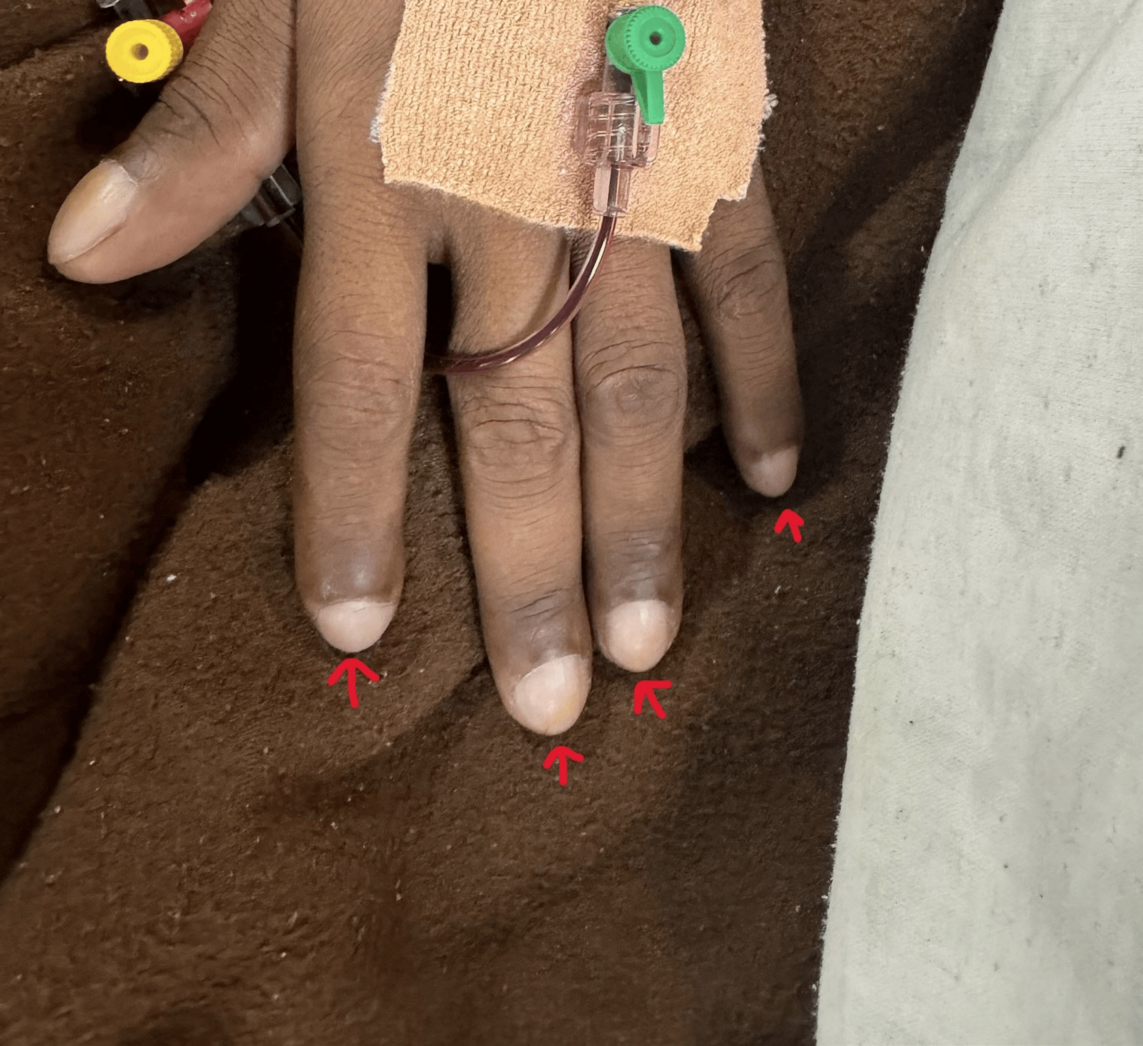
Grade 3 clubbing in the hand The image was taken by the authors

The examination of the vitals revealed the pulse rate to be approximately 84 beats per minute and a respiratory rate of 20 per minute. The patient weighed 48 kg, his height was 161 cm, and his BSA (body surface area) was 1.9 m^2^. The patient’s oxygen saturation was 71% (SpO_2_) on admission. On further examination, clubbing (grade 3), as shown in Figure 1, and increasing cyanosis were observed. On laboratory examination, hemoglobin was 16.2 g/dL, and hematocrit was 49.6. Serum sodium was 131 mE/L, and serum potassium was 3.4 mEq/L. Liver function tests and kidney function tests were normal.

**Figure 2:**
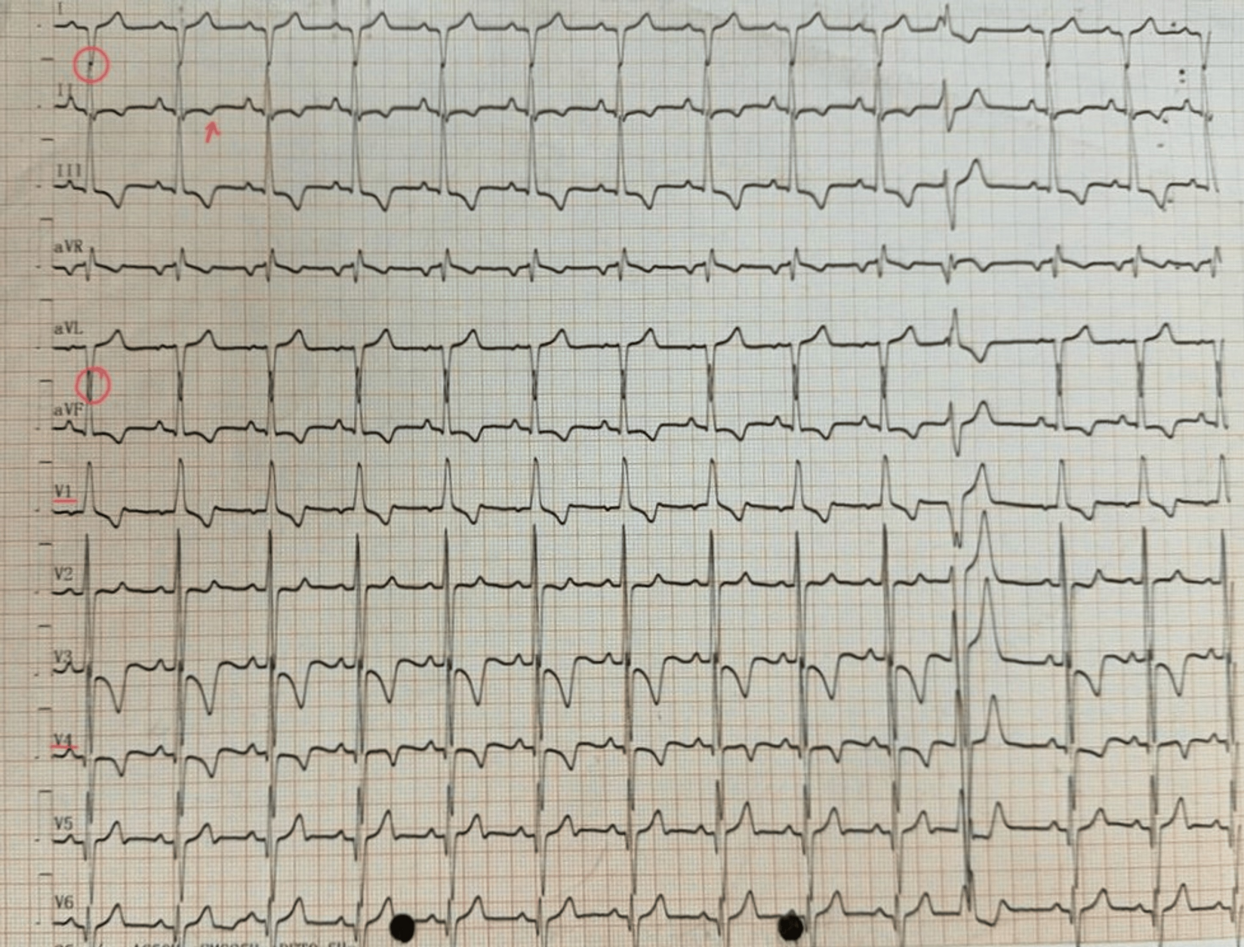
Electrocardiography showing right axis deviation in lead 1 and aVF (red circles), T-wave inversion (red arrow), and a transition of waves from lead V1 to V2 The image was taken by the authors aVF: augmented vector foot

The apex impulse was observed with a precordial bulge during the physical examination. Upon palpation, a thrill was detected at the apex beat over the fifth intercostal space and anterior axillary line. Auscultation revealed that S1 and S2 were best heard in the mitral area, accompanied by a pan systolic murmur. Electrocardiography (ECG) showed right axis deviation, and the right ventricle forces the R wave to the S wave, a transition from leads V1 to V2. T-wave inversion is also shown in Figure 2.

**Figure 3:**
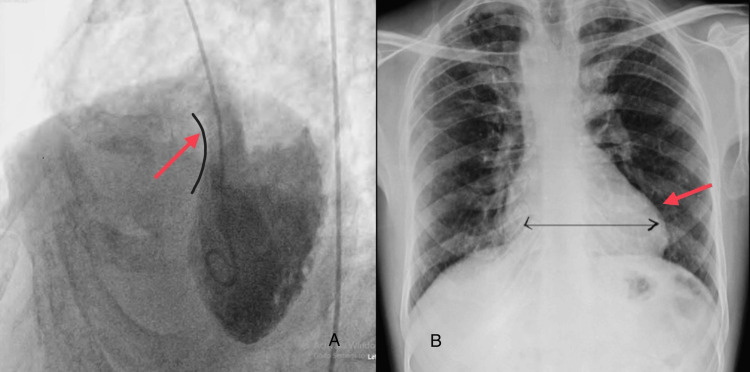
(A) The angiogram shows a malaligned sub-aortic ventricular septal defect (indicated by the red arrow pointing toward the black curve of the ventricle); (B) the postero-anterior view of the chest X-ray shows a boot-shaped heart with dilated ventricles (as marked by the red arrow) The images were taken by the authors

An angiogram was performed from the left ventricle (LV) to the aorta (AO), showing a smooth-walled, contractile posteriorly placed LV. LV morphology showed a large malaligned subaortic ventricular septal defect (VSD), as shown in Figure 3A, an opacifying right ventricle (RV) and pulmonary artery (PA). There is a severe sub-valvular and valvular pulmonary stenosis. A chest X-ray was taken, which revealed a boot-shaped heart (RV apex) and a right arch, as shown in Figure 3B.

A diagnostic cardiac catheterization was performed to assess the PA branches and MAPCAs. The access was made through the right femoral artery and the right femoral vein. A five-French sheath size was used. The pigtail and Judkins R catheters were used. The contrast dosage administered was 3 mL/kg. There were no associated complications. The sizes of various vascular structures are shown in Table 1.

**Table 1:** Z scores and measurements of major heart structures The table was created by the authors RPA: right pulmonary artery; LPA: left pulmonary artery; DAO: distal aorta; PA: pulmonary artery; RV: right ventricle; LV: left ventricle

Site	Measurements	Z score
RPA origin	12.4 mm	-
RPA distal	13 mm	-0.29
LPA origin	12 mm	-
LPA distal	12.4 mm	0.66
Pulmonary annulus	16 mm	-2.1
DAO	21 mm	3.65
Mc Goon	-	-
Mean PA pressure	27/14/18	-
RV	120/10	-
LV	120/10	-

**Figure 4:**
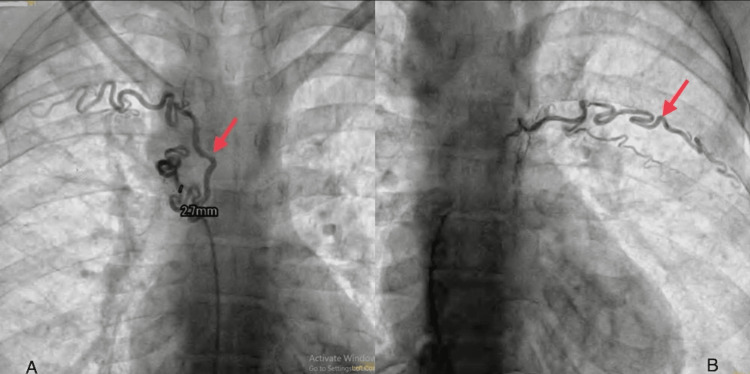
(A) Computed tomography pulmonary angiography showing a 2.7 mm MAPCA in the peri-tracheal area (marked by the red arrow); (B) angiogram showing MAPCA supplying the upper lung lobe (marked by the red arrow) The images were taken by the authors MAPCA: multiple aortopulmonary collateral artery

The computed tomography pulmonary angiography (CTPA) shows bilateral hypoplastic PAs. Multiple tortuous collaterals in peri-tracheal, peri-esophageal, and posterior mediastinum were observed. Multiple collaterals arising from descending aorta and left vertebral arteries were seen. A right coronary artery has a separate origin directly from the arch of the arch. MAPCAs measuring 2.7 mm are shown along with the fibrotic bands in apical segments of bilateral upper lobes are also observed, as shown in Figure 4A. A flush angiography was performed using a pigtail catheter course from the aorta to the RV. It revealed a 2.4-mm-long tortuous MAPCA, arising at the fourth thoracic (thoracic) spinal level supplying the left upper lung field with dual supply (Figure 4B).

**Figure 5:**
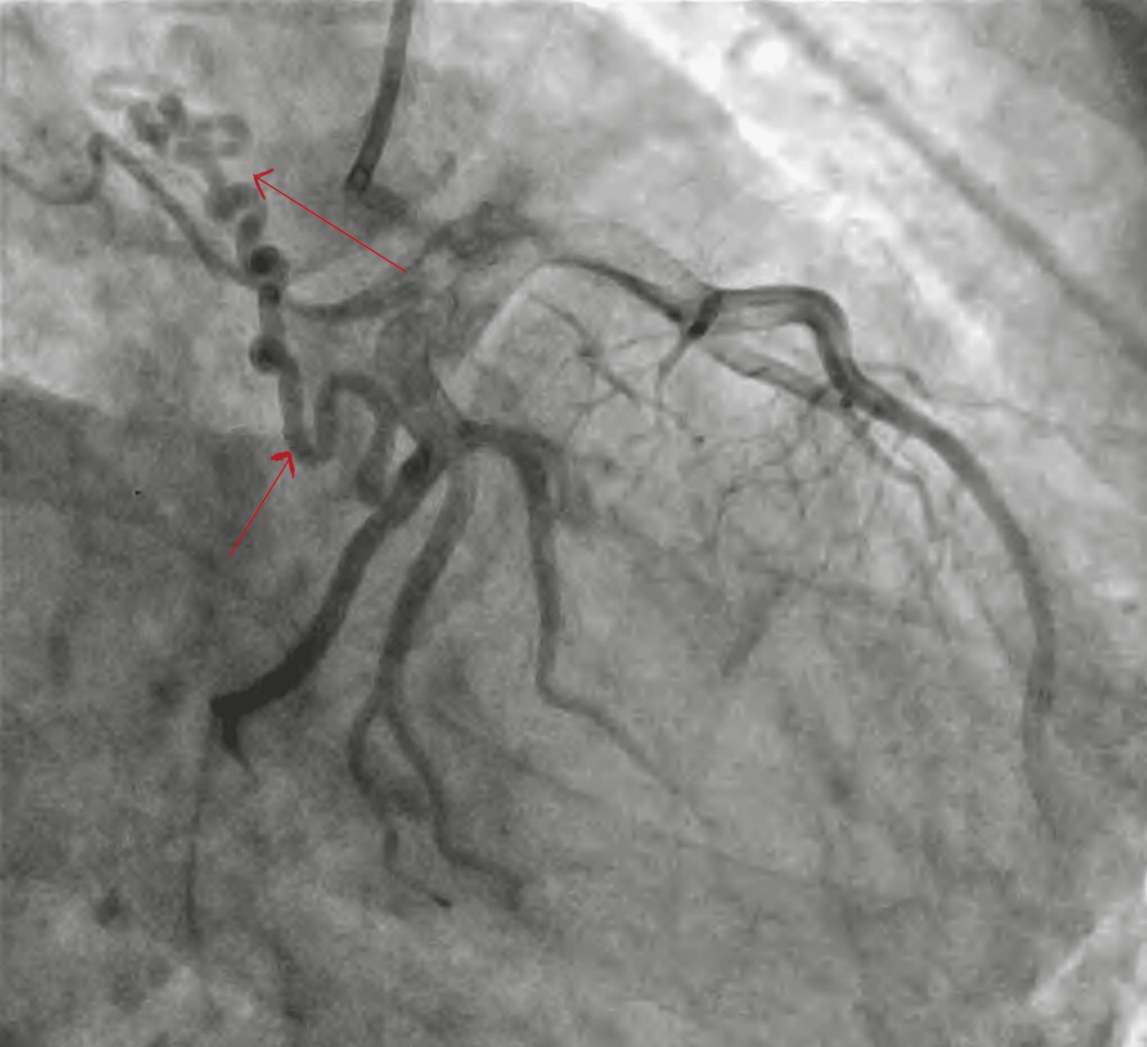
Angiogram showing coronary MAPCA and aplasia of the proximal left subclavian artery (as indicated by the red arrow) The images were taken by the authors MAPCA: multiple aortopulmonary collateral artery

The course from the RV to the inferior vena cava to the right atrium to the RV to the PA shows a coarsely trabeculated, well-contractile ventricle of RV morphology. There is severe sub-valvular and valvular pulmonary stenosis of confluent branch PAs. Aplasia of the proximal segment of the left subclavian artery, along with the coronary MAPCA, has been shown in Figure 5.

**Figure 6:**
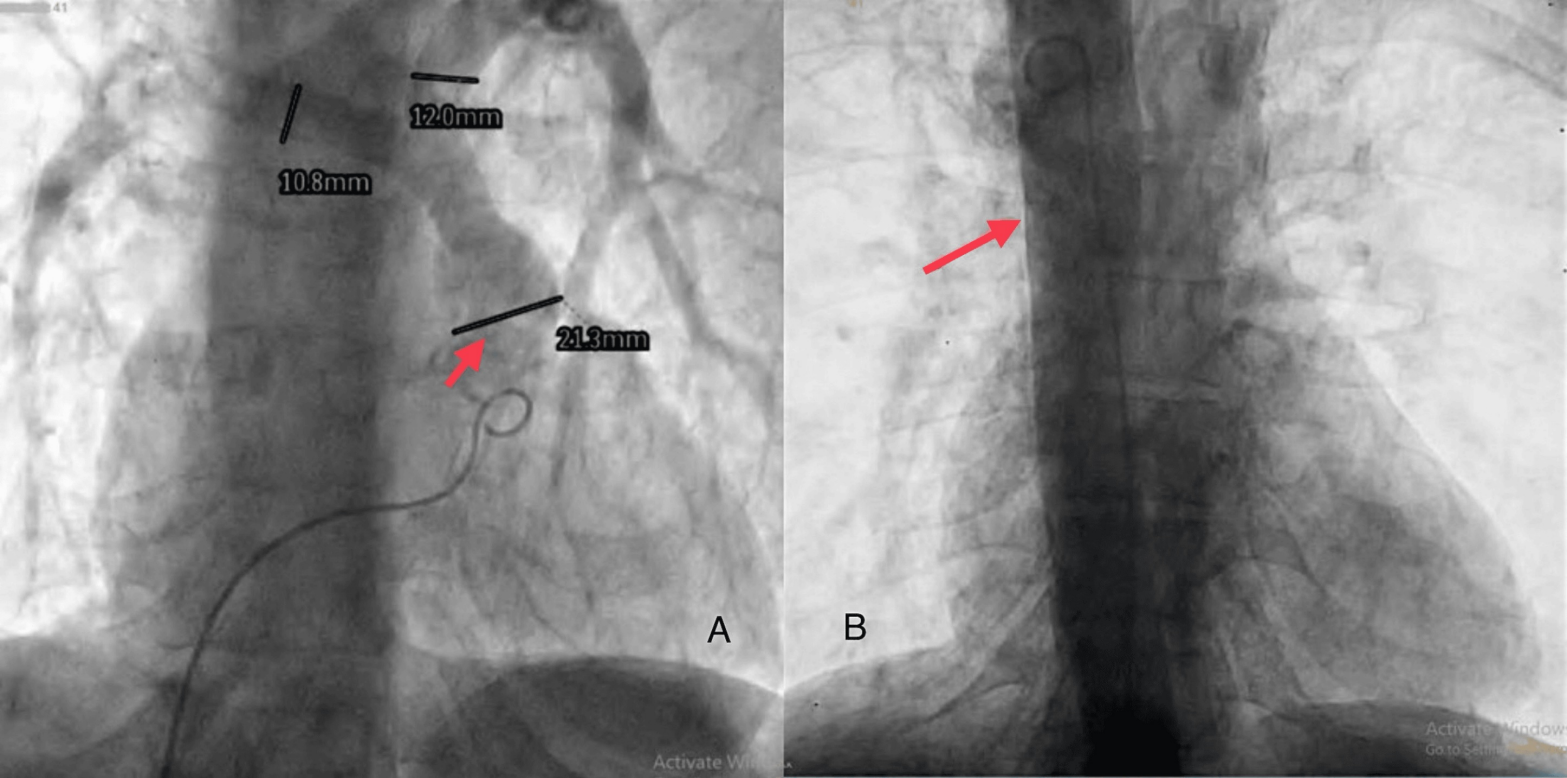
(A) Angiogram showing MAPCA (marked by the red arrow); (B) angiogram showing the right aortic arch (marked by the red arrow) The images were taken by the authors MAPCA: multiple aortopulmonary collateral artery

Angiography showed a coronary MAPCA measuring 2.1 mm, arising from left circumflex opacifying lung fields, as shown in Figure 6A. A flush angiogram was done using a pigtail catheter course from the aorta to the RV, showing the right aortic arch, as shown in Figure 6B, with a mirror image branching pattern of neck vessels. No patent ductus arteriosus or coarctation was seen.

The pre-cathlab diagnosis was CHD, which comprised of TOF features including a large malaligned nonrestrictive sub-aortic VSD (bidirectional shunts), aorta overriding present, severe infundibular and valvular PS, a confluent branch of the PA, right aortic arch, and normal biventricular function. The treatment plan was developed by a team of interventional cardiologists, cardiovascular surgeons, and interventional radiologists. The team decided that the patient should be managed with minimal invasive procedures, such as intrinsic cardiac repair (ICR) and transannular patch (TAP) with coil embolization of the MAPCAs. Additionally, the three lung MAPCAs were clipped and ligated with the pericardium. The gradient obtained in right ventricular outflow obstruction (RVOT) post-VSD closure was 80 mmHg, as detected by 2D echocardiography. After the procedure, the patient was completely well, and all his vitals were normal and stable. The patient was admitted to the intensive care unit (ICU) for observation. The patient is currently prescribed a tablet of fixar and a tablet of deriphyllin.

## Discussion

Assessing and evaluating all aspects of pulmonary circulation in TOF and MAPCA comprehensively poses a challenge. This challenge has significant implications for treating and investigating outcomes and risk factors [8]. In patients with TOF accompanied by pulmonary atresia and MAPCA, the risk of mortality is between 40% and 60% by the age of 1 and 10 years, respectively, if no intervention has been done [9]. Only a minority of individuals with TOF manage to reach adulthood without surgical repair, with only 3% surviving into their fourth decade [10]. As a result, cardiac surgery involving cardiopulmonary bypass (CPB) in adults with uncorrected TOF is extremely uncommon. However, successful aortic valve repair is achievable following aortic valve stenosis and complete TOF repair [11,12]. Only a limited number of aortic valve replacement cases in uncorrected TOF have been reported [13]. In our case, the patient is a middle-aged man without aortic valve involvement.

In a study by Fouilloux et al., out of 271 patients, they found DiGeorge syndrome in 110 patients with various MAPCAs. There are various MAPCAs concerning the place of origin and type of supply. With respect to the origin, the types are the descending thoracic aorta, right subclavian artery, left subclavian artery, coronary artery, transverse aortic arch, and abdominal aorta. With respect to the supply type, there is single supply, isolated supply to central PA, dual supply to central PA, and mixed supply [14]. In our case, three MAPCAs are seen to originate from the descending thoracic aorta, the left vertebral artery, and the left circumflex artery. Two surgical approaches are widely used for MAPCAs: one is the process that involves one or several steps of complete uni-focalization of the supplying MAPCAs, with or without integration of the native PAs. Additionally, it includes the connection of the RV to the newly formed pulmonary arteries (neo-PAs) and, if feasible, the simultaneous or subsequent closure of the VSDs. The other one is the rehabilitation of the innate PAs. Initially, a direct connection between the RV and the native PA is established to encourage the growth of the native PAs. This also facilitates interventional catheterization for dilatation or stenting of any stenosis and subsequent closure of communicant collaterals. Once the native PAs have adequately developed, the complete repair is undertaken. In cases where connecting a MAPCA to the PA is necessary (known as uni-focalization), the collateral artery is linked to an already-developed native branch [11].

## Conclusions

We present a case of multiple MAPCAs arising in peri-esophageal, peri-tracheal, and posterior mediastinal areas. MAPCAs originating from the descending aorta and left vertebral arteries have also been seen. Coronary MAPCAs with TOF and hypoplastic PAs together are rare cardiac manifestations, for which the treatment modalities are challenging. In our case, the patient was managed by a minimally invasive procedure using coil embolization and a transannular patch, with an RVOT gradient of 80 mmHg post-VSD closure. Post-procedural complications were nil, and the patient has a good postoperative prognosis.
